# Accumulation of GC donor splice signals in mammals

**DOI:** 10.1186/1745-6150-3-30

**Published:** 2008-07-09

**Authors:** Alexander Churbanov, Stephen Winters-Hilt, Eugene V Koonin, Igor B Rogozin

**Affiliations:** 1 Loyola University Medical Center, 2160 S. First Ave., Maywood, IL, 60153, USA; 2 Department of Computer Science, University of New Orleans, New Orleans, LA, 70148, USA; 3 National Center for Biotechnology Information NLM, National Institutes of Health, Bethesda, MD, 20894, USA

## Abstract

The GT dinucleotide in the first two intron positions is the most conserved element of the U2 donor splice signals. However, in a small fraction of donor sites, GT is replaced by GC. A substantial enrichment of GC in donor sites of alternatively spliced genes has been observed previously in human, nematode and Arabidopsis, suggesting that GC signals are important for regulation of alternative splicing. We used parsimony analysis to reconstruct evolution of donor splice sites and inferred 298 GT > GC conversion events compared to 40 GC > GT conversion events in primate and rodent genomes. Thus, there was substantive accumulation of GC donor splice sites during the evolution of mammals. Accumulation of GC sites might have been driven by selection for alternative splicing.

This article was reviewed by Jerzy Jurka and Anton Nekrutenko. For the full reviews, please go to the Reviewers' Reports section.

## Findings

In vertebrates, most of the protein-coding genes are interrupted by multiple introns that are removed at the donor and acceptor splice sites so that the adjacent exons are spliced. This process is mediated by an elaborate molecular machine, the spliceosome that consists of 5 snRNPs (small nuclear ribonucleoprotein particles) along with numerous less stably associated proteins, and is conserved throughout the eukaryotic world [1-3]. The U2 spliceosome (the major eukaryotic spliceosome) interacts with specific parts of the intron and the flanking exons to ensure accurate and efficient splicing [4]. The nucleotides at the intron termini and the adjacent nucleotides in the exons are involved in these interactions and comprise the splicing signal. The (A/C)AG|GT(A/G)AGT consensus sequence (the exon|intron boundary is shown by the vertical streak and the first two nucleotides of the intron are underlined) at the donor splice signal is complementary to the 5' end of U1 snRNA, and this interaction is believed to be the major requirement for splicing [5-7].

The GT dinucleotide in the first two intron positions is the most conserved element of the U2 donor splice signal. However, in a small fraction of donor sites (<1%), GT is replaced by GC; in these cases, the rest of the nucleotides in the donor signal adhere more closely to the consensus sequence, apparently, compensating for the T to C substitution that is unfavorable for splicing [8-10]. This rare class of donor splice signals has been implicated in alternative splicing [9,11,12]. For example, the conserved C at the +2 position of the 10^th ^intron of the let-2 gene which encodes one of the collagen isoforms is essential for developmentally regulated alternative splicing in the nematode *C. elegans*. Replacement of the GC donor signal with a moderate or strong GT signal abolishes splicing regulation and leads to excessive usage of exon 10 of let-2 in embryos [11]. Generally, a substantial enrichment of GC donor signals in alternatively spliced genes has been observed in human, *C. elegans *and Arabidopsis [9,11,12].

Pairwise comparisons of GC splicing signals in the nematodes *Caenorhabditis elegans *and C. *briggsae *suggested that GC donor signals are not evolutionarily conserved in nematodes: among the 26 *C. elegans *GC-AG introns, only 5 had a GC-AG counterpart in C. *briggsae *[11]. Frequent switching between GT-AG and GC-AG introns has been reported for 5 vertebrate genomes [10]. We were interested in exploring the genome-wide evolutionary dynamics of the donor splice sites and, in particular, sought to determine whether there might be a trend toward depletion or accumulation of GC.

**Figure 1:**
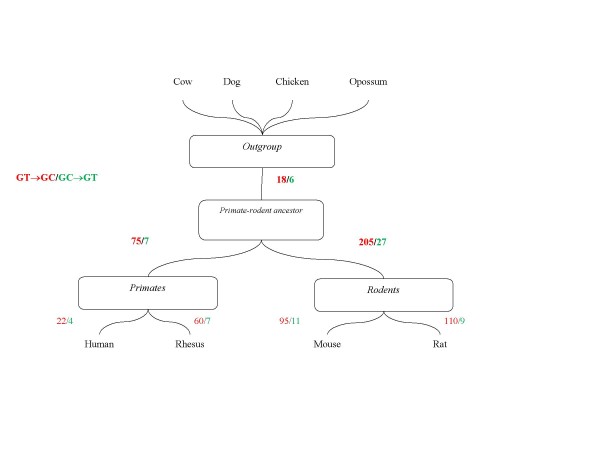
**Parsimony reconstruction of GT > GC (red) and GC > GT (green) donor signal conversion events mapped on the phylogenetic tree of 8 vertebrate species**. The tree topology that includes the primate-rodent clade was from [16].

Genomic alignments of 8 vertebrates (chicken, opossum, dog, cow, rhesus macaque, human, mouse and rat) were extracted from the UCSC genome browser (Additional file 1) [13,14] and used to map cases of GC > GT and GT > GC conversion to the branches of the mammalian phylogeny (Figure 1). Terminal leaves of the tree (individual genomes) are more vulnerable to the effects of sequencing errors and/or population polymorphism than internal branches [15]. Therefore, only those sites were analyzed in which the GC or GT donor signal was shared by at least two sister taxa (e.g., we assign the GC signature to the rodent clade when GC was found in both mouse and rat sequences). It was further required that either GT or GC signal was conserved in all outgroup species for which an alignment was available at the given site (the presence of the dog and cow sequences was unconditionally required). Altogether, there were 122,621 and 253 invariant GT and GC donor signals, respectively, and 656 variable sites of which 338 mapped to internal branches and were employed for the analysis of evolutionary dynamics.

The GC>GT and GT>GC conversion events were reconstructed using maximum parsimony (Figure 1). Unexpectedly, we observed a pronounced excess of GT>GC conversion over GC>GT conversion that is indicative of accumulation of GC donor splice sites in both primate and rodent genomes (Figure 1). The trend is stronger in the rodent lineage than in the primate lineage (Figure 1), an observation that is consistent with the overall fast genome evolution in rodents [16]. The apparent accumulation of GC donor signals was further supported by the analysis of the terminal branches of the tree although the excess of GC>GT conversions in macaque compared to human (Figure 1) could be caused by sequencing errors and/or population polymorphism. The observed excess of GT>GC conversions was robust with respect to the composition of the outgroup species set (Additional file 2).

The observed excess of GT>GC conversions hardly can be explained by a nucleotide substitution bias. It has been repeatedly shown that mammalian genomes have a tendency to become more AT-rich [17-19]. Even if one assumes that, due to unknown reasons, this trend is reversed in the donor sites so that T to C substitutions are twice as frequent as C to T substitutions, such a bias would not account for the observed excess of GT>GC conversion events (P < 10^-10 ^according to the χ^2 ^test for pooled conversion events).

Considering that mutational bias did not seem to be a plausible cause of the observed accumulation of GC donor sites, it seems most likely that this trend has to do with the involvement of GC sites in alternative splicing that is widespread and essential in mammals [9,11,12]. As GT>GC conversion can substantially alter the pattern of alternative splicing [11], these changes might become beneficial and eventually would be fixed in the population. Thus, positive selection could be a plausible explanation for the observed accumulation of GC in donor sites. However, an even more plausible scenario would involve evolution of a strong splice site context that would allow neutral fixation of GC sites. The neutrally fixed GC sites, then, could be recruited for alternative splicing and thus would become subject to purifying selection forbidding the reverse GC>GT conversion.

## Competing interests

The authors declare that they have no competing interests.

## Authors' contributions

AC performed sequence comparisons, SWH contributed to the interpretation of the results, AC and IBR contributed to the analysis of the results and wrote the initial draft of the manuscript, IBR and EVK incepted the study, contributed to the analysis of the results and wrote the final manuscript, all authors edited and approved the final version.

## Reviewers' comments

### Reviewer's report 1: Jerzy Jurka, Genetic Information Research Institute

This paper reports the relatively straightforward observation that GC donor splice sites tend to accumulate during evolution of mammals. However, the suggestion of selection for alternative splicing would be more convincing if the authors could demonstrate the GC accumulation separately in AT-rich and GC-rich genomic regions in mammals, where the dynamics of GT replacement by GC may be different.

#### Authors' response

*We appreciate the suggestion that the dynamics of GC accumulation could depend on the base composition in the respective regions of the mammalian genomes*.

*So we performed a crude comparison of the occurrence of GC donor sites and the rates of donor site conversion in AT-rich and GC-rich regions*. Table 1*shows that, although there was a statistically significant excess of GC donor sites in GC-rich regions, there was only a marginal, not statistically significant difference between the corresponding rates of donor site conversions. Thus, it appears that the accumulation of GC donor sites described here does not strongly depend on the nucleotide composition of the corresponding genomic regions*.

**Table 1:** GT and GC donor splice sites and conversion events in AT-rich and GC-rich regions^a^

Splice sites/conversion events	%A+T > 50%	%A+T < 50%	P_Fisher_/GT^b^
Total number of donor splice sites(%)	51798(100)	71396(100)	
GT	51575(99.57)	71028(99.48)	
GC	88(0.17)	165(0.23)	0.02
GT>GC	113(0.22)	185(0.26)	0.15
GC>GT	22(0.04)	18(0.03)	0.11

^a^AT content was estimated in ± 100 base pair regions surrounding donor splice sites

^b^Statistical significance was estimated by comparing the respective values with the number of GT sites using Fisher's two-tailed test

### Reviewer's report 2: Anton Nekrutenko, Pennsylvania State University

In this discovery note authors point out accumulation of non- canonical GC donor splice signals in mammals, against the previously observed nucleotide substitution bias. They provide a convincing explanation suggesting that GT->GC conversion may be beneficial for mammals as it creates additional possibilities for alternative splicing events. In my opinion these observations provide a platform for launching more detailed investigation of alternative splicing through comparative genomics and raise numerous interesting question (e.g., are any of the GC sites overlap with known SNPs?). Thus publication of this note will appeal to a broad evolutionary genomics community.

On a technical side the authors used a rather complex procedure for retrieving splice sites from TBA alignments. Instead, this can be easily and quickly done using Galaxy system:



as explained here:



#### Authors' response

*We appreciate the reviewer pointing out the utility of the Galaxy platform and hope to exploit Galaxy in future genome analyses*.

## Supplementary Material

Additional file 1 Materials and Methods. Click here for file

Additional file 2 Parsimony reconstruction of GT > GC and GC > GT donor signal conversion events for different sets of outgroup species. Click here for file
