# Supplementary material for: Accumulation of GC donor splice signals in mammals
Source: Biol Direct. 2008 Jul 9;3:30. doi: 10.1186/1745-6150-3-30 (PMC2490688; doi:10.1186/1745-6150-3-30)
Supplement: Additional file 1 — Materials and Methods. [file 1745-6150-3-30-S1.doc]

# Accumulation of GC donor splice signals in mammals

Alexander Churbanov^1^, Stephen Winters-Hilt^2^, Eugene V. Koonin^3^, Igor B. Rogozin^3^

^1^Loyola University Medical Center, 2160 S. First Ave., Maywood, IL 60153, USA; ^2^Department of Computer Science, University of New Orleans, New Orleans, LA 70148;

^3^National Center for Biotechnology Information NLM, National Institutes of Health, Bethesda, MD 20894, USA

**Additional file 1**

Materials and Methods

Multiple alignments of 17 vertebrate genomes were downloaded from the UCSC genomic browser (http://genome.ucsc.edu/) [1]. The “threaded blockset alignments” [2] were projected onto human reference exons predicted by aligning human reference sequences (<ftp://ftp.ncbi.nih.gov/refseq/H_sapiens/mRNA_Prot>) against human chromosomal assemblies (<http://hgdownload.cse.ucsc.edu/goldenPath/hg18/chromosomes/>) using the BLAT program [1] that has been shown to produce one of the most accurate predictions of mammalian gene structure [3].

The procedure included the following steps:

1. Remove all reference sequences with accession number not starting with `NM_' (such as computationally predicted transcripts).
2. Eliminate gaps shared by all 8 analyzed species (chicken, opossum, dog, cow, rhesus macaques, human, mouse and rat) within segments in the multiple alignment blocks.
3. Align human reference sequences and human chromosome assemblies using BLAT. Parse BLAT alignments. Remove all spliced alignments with average accuracy less than 99% coverage.
4. Extract overlaps between genomic multiple alignments and predicted exons in the human genome and define exon/intron boundaries in multiple alignments.
5. Parse corresponding chromosomal assemblies to extend each species nucleotide segment in the block and confirm the presence of U2 splice signals using Bayesian splice site sensor [4].

Using this procedure, 158,263 donor sites were mapped on 16,105 human gene structure predictions. Using the identified human splice signals as the guide, 1,103,062 vertebrate donor signals were extracted from the aligned sequences.

# References

1. Kuhn RM, Karolchik D, Zweig AS, Trumbower H, Thomas DJ, Thakkapallayil A, Sugnet CW, Stanke M, Smith KE, Siepel A *et al*: **The UCSC genome browser database: update 2007**. *Nucleic Acids Res* 2007, **35**(Database issue):D668-673.

2. Blanchette M, Kent WJ, Riemer C, Elnitski L, Smit AF, Roskin KM, Baertsch R, Rosenbloom K, Clawson H, Green ED *et al*: **Aligning multiple genomic sequences with the threaded blockset aligner**. *Genome Res* 2004, **14**(4):708-715.

3. Churbanov A, Pauley M, Quest D, Ali H: **A method of precise mRNA/DNA homology-based gene structure prediction**. *BMC Bioinformatics* 2005, **6**:261.

4. Churbanov A, Rogozin IB, Deogun JS, Ali H: **Method of predicting splice sites based on signal interactions**. *Biol Direct* 2006, **1**:10.
