# Supplementary material for: Accumulation of GC donor splice signals in mammals
Source: Biol Direct. 2008 Jul 9;3:30. doi: 10.1186/1745-6150-3-30 (PMC2490688; doi:10.1186/1745-6150-3-30)
Supplement: Additional file 2 — Parsimony reconstruction of GT > GC and GC > GT donor signal conversion events for different sets of outgroup species. [file 1745-6150-3-30-S2.doc]

# Accumulation of GC donor splice signals in mammals

Alexander Churbanov^1^, Stephen Winters-Hilt^2^, Eugene V. Koonin^3^, Igor B. Rogozin^3^

^1^Loyola University Medical Center, 2160 S. First Ave., Maywood, IL 60153, USA; ^2^Department of Computer Science, University of New Orleans, New Orleans, LA 70148;

^3^National Center for Biotechnology Information NLM, National Institutes of Health, Bethesda, MD 20894, USA

**Additional file 2**

Parsimony reconstruction of GT > GC and GC > GT donor signal conversion events for different sets of outgroup species.

Cow

Dog

Chicken

Opossum

Human

Rhesus

Mouse

Rat

Primate-rodent ancestor

*Outgroup*

*Primates*

*Rodents*

**11/1**

**44/1**

**124/9**

50/6

43/3

29/3

12/0

**GT→GC/GC→GT**

**Parsimony reconstruction of GT > GC (red) and GC > GT (green) donor signal conversion events mapped on the phylogenetic tree of 8 vertebrate species for 4 outgroup species.** The total number of donor splice signals with invariant GT and GC donor signals was 74,121 and 131, respectively.

Cow

Dog

Chicken

Opossum

Human

Rhesus

Mouse

Rat

*Primate-rodent ancestor*

*Outgroup*

*Primates*

*Rodents*

**16/3**

**65/2**

**175/24**

93/9

80/9

53/5

20/0

**GT→GC/GC→GT**

**Parsimony reconstruction of GT > GC (red) and GC > GT (green) donor signal conversion events mapped on the phylogenetic tree of 8 vertebrate species for 3 outgroup species.** The total number of donor splice signals with invariant GT and GC donor signals was 112,977 and 213, respectively.
